# Coagulation Assessment and Fibrinogen Thresholds in Therapeutic Plasma Exchange—A Narrative Review

**DOI:** 10.3390/biom16091304

**Published:** 2026-09-09

**Authors:** Matej Zrimsek, Jakob Gubensek

**Affiliations:** 1 Department of Nephrology, University, Medical Centre Ljubljana, Zaloska Cesta 7, 1000 Ljubljana, Slovenia; matej.zrimsek@kclj.si; 2 Faculty of Medicine, University of Ljubljana, Vrazov Trg 2, 1000 Ljubljana, Slovenia

**Keywords:** bleeding risk assessment, bleeding complications, dilutional coagulopathy, factor XIII, fibrinogen, fibrinogen concentrate, fresh frozen plasma, rotational thromboelastometry (ROTEM^®^), apheresis, therapeutic plasma exchange

## Abstract

Many therapeutic apheresis procedures that remove plasma or plasma components also lower coagulation factor levels, which can increase patients’ bleeding risk. In this narrative review, we sum up data on clotting factor removal and the development of dilutional coagulopathy mostly associated with therapeutic plasma exchange (TPE) treatment and consider available tests for coagulation assessment. It is common in clinical practice to check fibrinogen levels regularly during repetitive TPE treatments and use it as a marker of hemostasis because of its long half-life and central role in hemostasis. We discuss the background of current clinical practice regarding fibrinogen thresholds discuss different threshold levels and data on the associated risk of bleeding. We stress the importance of additional bleeding risk assessment when patients are exposed to concomitant invasive procedures. We also describe the possibilities for fibrinogen replacement during TPE treatments with either fresh frozen plasma or fibrinogen concentrate and the associated effect on factor XIII levels. Finally, we briefly discuss alternative apheresis procedures regarding their fibrinogen-sparing potential.

## 1. Introduction

Therapeutic apheresis encompasses different extracorporeal procedures used to treat patients by separating and removing disease-causing blood components. Most diseases treated with apheresis are autoimmune in nature and are mediated by plasma constituents, most commonly immunoglobulins. Although selective apheresis techniques, capable of specifically removing immunoglobulins or other target plasma molecules, are available, nonselective therapeutic plasma exchange (TPE) remains the most commonly performed apheresis procedure [1,2,3]. During TPE, the patient’s plasma is removed and replaced with a replacement solution. The indications for the use of TPE are broad and are summarized in current American Society for Apheresis guidelines [4].

The preferred replacement solution for most diseases treated with TPE is an albumin solution [4]. Only in a few diseases, where replacement of certain plasma constituents is desired, plasma acquired from healthy donors is used as a replacement solution. The use of albumin solution lowers the risk of adverse events associated with plasma and can reduce the cost of the procedure in some countries, while it can be more expensive in others [4,5,6]. Since albumin solutions contain no coagulation factors, their concentration is lowered during the course of TPE, leading to dilutional coagulopathy. This effect becomes more pronounced when short intervals between procedures do not allow sufficient time for complete hepatic restoration of coagulation factors [7,8].

Most commonly, one plasma volume is exchanged during a single TPE procedure, which reduces the concentration of substances in the intravascular compartment during the procedure by approximately 63%, although the degree of reduction can differ among individual molecules [9,10]. The primary therapeutic target for removal is most often a pathological antibody, predominantly immunoglobulin G (IgG), but the procedure also removes coagulation factors and inhibitors, albumin and many other plasma components, platelets and cytokines (which can also promote coagulation) [11]. Since less than half of IgG is intravascular, repetitive TPE procedures are performed at short intervals to achieve a sufficient reduction in IgG concentration and the desired clinical response. Procedures are typically performed daily to every other day depending on the indication and urgency [4,7,8]. When albumin is used as a replacement solution, a dilutional coagulopathy develops and temporarily increases bleeding risk. Fibrinogen and factor XIII exhibit the slowest reconstitution rates and may therefore reach the lowest concentrations with repeated TPE procedures, which should be considered when planning treatment [8,12,13]. Consequently, fibrinogen is most commonly used as a marker of hemostasis in apheresis medicine and the threshold of 1.0 g/L before TPE is mostly used as a cut-off below which coagulation factor replacement during TPE is considered to lower the risk for bleeding events [14,15].

In this review, we discuss the background for the development of dilutional coagulopathy associated with TPE treatment, consider possible coagulation assessment options, discuss the background of current clinical practices regarding fibrinogen thresholds and describe the possibilities for fibrinogen replacement during TPE treatments. Finally, we also briefly discuss alternative apheresis procedures regarding their fibrinogen-sparing efficiency.

## 2. Removal of Clotting Factors and Development of Dilutional Coagulopathy

Individual coagulation factors differ substantially in their half-lives and recovery times following a single TPE procedure. Our knowledge on removal and kinetics of clotting factors is based on studies performed about 50 years ago, when apheresis practices differed somewhat from current clinical practice [10,11,12,13,16,17,18]. Most of these studies used a fixed exchange volume without adjustment for individual patient plasma volume and predominantly used heparin as the anticoagulant. Chirnside et al. used heparin anticoagulation with a centrifugal plasma separator and exchanged plasma volumes of 4000 mL with coagulation factor-free replacement fluid in six patients. They measured fibrinogen, thrombin, factors V, VII, VIII, IX, X and antithrombin III before, immediately after and days after TPE to determine recovery of coagulation factors. Factors V, VII, VIII, IX and antithrombin III levels recovered well already after 24 h, while recovery of other factors was slower (see Table 1). Slow recovery of fibrinogen and factor X levels was observed and after 2 days their levels were within the normal range in only 52% and 56% of cases [10].

Flaum et al. also exchanged a fixed plasma volume of 4000 mL and used heparin for anticoagulation. Their observations regarding the recovery of coagulation factors were largely comparable to those reported by Chirnside et al., with the exception of factor X, for which recovery to normal levels was observed within 24 h, but these data refer to a single patient [17]. An almost complete recovery of factor X after 48–96 h after TPE is reported in a larger cohort by Sultan et al., exchanging 160% of the patient’s plasma volume [16]. Wood et al. exchanged a pre-calculated plasma volume corresponding to 1.5 times the patient’s estimated plasma volume and used citrate anticoagulation. In their study, recovery of factor VIII and antithrombin III was somewhat slower, although their concentrations also normalized within 48 h. In comparison, normalization of fibrinogen levels required up to 72 h. In these older studies, factor XIII measurement was not yet available, but newer data show that it also has a slow restitution rate and can reach quite low levels, e.g., 20% before and below 10% after repeated apheresis [7,19,20].

While most studies analyzing coagulation factor removal and restitution were conducted with centrifugal plasmapheresis, similar results should be expected with single-filtration plasmapheresis [21].

In summary, fibrinogen and factor XIII exhibit the slowest reconstitution rates and may therefore reach the lowest concentrations with repeated TPE procedures. Fibrinogen acts as a precursor of fibrin and ultimately prevents bleeding and its slow restitution time after TPE can make it a limiting factor. Factor XIII is responsible for cross-linking fibrin strands and stabilizing the clot. However, its contribution to bleeding risk is generally considered less clinically significant [7,8,22], and its measurement is usually not available in routine clinical practice. Therefore, pre-procedure fibrinogen has traditionally been used to determine if TPE can be safely performed without coagulation factor replacement.

## 3. Assessing the Severity of Dilutional Coagulopathy

Observational data on inherited single-factor deficiencies show that quite low individual factor levels (15–20% activity) can be tolerated without excessive risk of spontaneous bleeding. Table 2 summarizes selected coagulation factors and their minimum hemostatic levels as reported by Mannucci et al. [23]. These hemostatic levels are based on limited data on bleeding episodes and refer to individual factor deficiencies and should therefore be interpreted with caution in the setting of multi-factor deficiency in dilutional coagulopathy induced by TPE. Nevertheless, they probably can represent an approximate absolute lowest acceptable level of individual factors to prevent spontaneous bleeding. Since it is difficult to measure concentrations of most coagulation factors on a daily basis and each factor individually usually does not impose a clinically significant coagulopathy during routine TPE, such measurements are not used in everyday clinical practice.

For global assessment of hemostasis, standard coagulation tests such as activated thromboplastin time and prothrombin time are readily available and are typically prolonged immediately after TPE but generally normalize within 4 to 24 h following the procedure. Moreover, the degree of prolongation is usually modest [12,16,17], and their ability to predict overall or TPE-associated bleeding risk is limited [24,25] and can even be inferior to a detailed patient bleeding history [26,27], since standard coagulation tests were created to screen for specific abnormalities or guide treatment with anticoagulant medication and can have a high specificity but low sensitivity [28,29,30]. Mild bleeding disorders can only be diagnosed by detailed patient and family history with the help of diagnostic tools, followed by specific laboratory testing [30].

In addition to standard coagulation assays, rotational thromboelastometry (ROTEM^®^) has also been evaluated in patients undergoing TPE [31,32,33]. Such testing may be particularly useful in patients with active bleeding or in those scheduled for surgical procedures, where it can help guide decisions regarding fibrinogen and factor XIII replacement because standard coagulation tests are not very sensitive for lower fibrinogen values and fail to show any factor XIII deficiency [34,35]. As a point-of-care bedside method, ROTEM^®^ may also be more readily available than measurement of fibrinogen level, let alone the factor XIII activity [36,37,38,39,40], but there are no data from larger cohorts on its ability to predict bleeding in a TPE setting.

Given all the limitations of other tests, fibrinogen remains the only parameter routinely monitored during TPE in clinical practice because of its long half-life, central role in hemostasis, and widespread availability of laboratory testing. Although factor XIII also has a prolonged half-life, its activity likely correlates reasonably well with the more easily measurable fibrinogen concentration [41]. Although fibrinogen has traditionally been used in apheresis medicine since its inception to exclude severe bleeding risk, the working group of the American Society of Apheresis following the Choosing Wisely campaign recently advised against measuring any coagulation tests during a course of TPE when using 1.0 or even 1.5 plasma volume exchanges, unless the procedure is performed daily [42]. This recommendation was based on the above-mentioned evidence suggesting that coagulation factor activities of approximately 15–20% are generally sufficient to prevent spontaneous bleeding [23,43]. While those recommendations can probably simplify clinical routine in stable patients without bleeding risk, it should be noted that patients who have a bleeding risk present (e.g., post-procedure or receiving anticoagulation treatment) should still be monitored with fibrinogen levels.

## 4. Fibrinogen Thresholds and Clinical Bleeding

The optimal fibrinogen threshold remains a matter of debate; nevertheless, a 1.0 g/L threshold is likely the most universally accepted cut-off below which coagulation factors are replaced during TPE. The evidence supporting this clinical practice is not very strong. Most likely, it results from the fact that a pre-TPE level of about 1.0 g/L results in post-TPE levels of about 0.5 g/L, which are considered critical for spontaneous bleeding in patients with congenital afibrinogenemia [23]. Furthermore, fibrinogen levels approaching 0.5 g/L (after TPE) are associated with INR values ≥ 1.5 [20], and an INR value below 1.5 is usually required for complete reversal of anticoagulation prior to surgery [44,45]. Clinically significant bleeding during a course of TPE is very rare, unless the patient has other factors contributing to an increased bleeding risk [46,47,48].

Data on the association between fibrinogen levels and bleeding in patients on apheresis are very diverse. Historically, apheresis specialists were quite brave. Keller et al. report a study on patients with TPE without coagulation factor replacement [18]. The study included 179 TPE procedures performed in 18 patients using exchanged volumes of 4000 or 5200 mL. Heparin was used for anticoagulation, followed by protamine sulfate administration after the procedure to reverse its effect. Although interprocedural intervals varied among patients, several underwent consecutive daily TPEs. Among six patients evaluated after their fifth consecutive daily TPE, fibrinogen concentrations decreased to a mean of only 11% of baseline values. One patient even underwent 10 consecutive TPE procedures, after which the fibrinogen concentration declined to only 1% of the baseline; during the last few TPEs, the fibrinogen must have been <0.1 g/L, although the exact level is not given in the original publication. Despite these profound reductions in fibrinogen levels, bleeding complications occurred in only three patients, corresponding to an incidence of 2.2% per TPE procedure. All three patients with bleeding had pre-TPE fibrinogen > 1.3 g/L (i.e., in line with current practice), and in all three cases, bleeding was attributed to low platelet count, which was under 30 x 10^9^/L in all cases. Bleeding was successfully managed with transfusion of six platelet concentrates per patient, without administration of fresh frozen plasma (FFP). In this study, the mean post-TPE platelet count for all patients was only 50%, 21% and 23% of baseline values after the first, fifth, and tenth consecutive daily TPEs, which may have been caused by suboptimal procedure settings at that time and partially by the concurrent immunosuppression.

Sultan et al. observed no bleeding episodes in 7 patients with heparin and citrate anticoagulation during TPE with a mean of 1.6 plasma volumes exchanged. On the contrary, they even reported two thrombotic episodes, one deep vein thrombosis at the catheter site and one case of clotting within the circuit, which were partially attributed to the increase in factor VIII and mostly to the decrease in antithrombin III values during TPEs [16]. As was later commented by Chirnside et al., low antithrombin III levels could be caused by consumption and not only its removal [10], which can also be partially procedure-related.

While thrombotic episodes are possible and are often associated with central venous catheters, contemporary studies often report bleeding episodes. They are usually mild and predominantly vascular-access related. Importantly, most patients who experienced bleeding had concomitant thrombocytopenia or were on anticoagulant therapy. Nevertheless, clinically significant bleeding episodes do occur, and even fatal bleeding complications have been reported in exceptional cases [47,48,49]. While there are reports that bleeding may affect nearly 20% of patients in certain cohorts [48], the percentage calculated per procedure is mostly below 3% [50,51]. Recently, Raval et al. published data on patients undergoing up to five consecutive daily TPE procedures without routine coagulation factor replacement or fibrinogen monitoring [52], similarly to the aforementioned article by Keller [18]. Despite intensive TPE, the bleeding rate was 1.4% per procedure and 6.1% per patient, with most bleeding events related to vascular access. Some additional details from the aforementioned studies are summarized in Table 3.

Regardless of these somewhat extreme reports on intensive TPE without fibrinogen monitoring, the fibrinogen threshold of 1.0 g/L is commonly followed, as discussed above. However, Wodayo et al. recently reported favorable outcomes after lowering this threshold to 0.8 g/L in patients without significant bleeding risk. In their study, only 3% of patients experienced minor bleeding complications at the vascular access site, and even those were concomitantly receiving direct oral anticoagulants [53], which, in our opinion [54], should also be considered a bleeding risk.

Thrombotic complications were most often reported in hospitalized patients with limited mobility. Both thrombotic and bleeding complications are frequently associated with central venous catheters used for vascular access during TPE treatment [16,47,55]. Therefore, prophylactic anticoagulation is likely warranted in most hospitalized (acutely or critically ill) patients undergoing TPE who are not on anticoagulants, as most of them have many risk factors for thrombotic events [56].

## 5. Assessing the Patient’s Bleeding Risk

Estimating the risk of bleeding due to dilutional coagulopathy induced by TPE in an individual patient is difficult. As mentioned previously, standard coagulation tests are usually only mildly affected during TPE treatment and correlate poorly with bleeding risk. In addition to the patient’s natural bleeding tendency and severity of dilutional coagulopathy, the severity of any invasive procedure, which has been performed or is planned, must also be considered and might be the most important factor affecting the bleeding risk.

There are no guidelines designed specifically for patients on TPE, but estimated risks of different invasive procedures can be transferred from recommendations guiding anticoagulant treatment, where more clinical data are available [45]. Patients treated with TPE commonly undergo procedures such as native or transplanted kidney biopsy, lumbar puncture, or kidney transplantation. All those procedures are classified as procedures with high bleeding risk (see Table 4 [57]) at the time of the procedure, while the bleeding risk then slowly reduces after some time (a few days). Patients can also have disease-related thrombocytopenia or bleeding complications, e.g., gastrointestinal bleeding.

Recommendations for patients receiving anticoagulant treatment are not fully transferable to the TPE setting, because these patients need to be anticoagulated to prevent thromboembolic events, while patients treated with TPE do not “need” the dilutional coagulopathy imposed on them by TPE treatment, although the TPE itself is necessary. Therefore, the risk-benefit ratio changes. While the thrombotic risk is low, the bleeding risk can be high in patients after (or prior to) invasive procedures or those on anticoagulant therapy. Therefore, the most important factor determining the depth of dilutional coagulopathy that can be tolerated in a certain patient is his bleeding risk, and most likely higher fibrinogen levels need to be maintained to prevent excessive prolongation of clotting tests.

## 6. Concomitant Anticoagulation Therapy

Patients requiring therapeutic anticoagulation during the course of TPE treatment represent a specific challenge. Prescription of anticoagulant therapy during TPE is complicated by the fact that TPE removes not only coagulation factors but also target molecules (e.g., antithrombin III) required for the activity of certain anticoagulants, as well as the anticoagulant drugs themselves [58]. We have data showing a doubling of INR during TPE with albumin in stable patients on warfarin [59]. In summary, the effect of anticoagulants in the presence of dilutional coagulopathy is hard to predict and monitor, especially when using drugs without a simple and readily available laboratory test.

Given all the unknowns, it seems prudent to use short-acting anticoagulants (i.e., standard heparin) and to maintain higher fibrinogen levels after TPE. Using a fibrinogen threshold of 1.5 g/L for execution of TPE with albumin will prevent excessive drops (<0.5 g/L) during TPE, while replacing fibrinogen during TPE in those with lower levels is necessary to maintain a post-TPE level above 0.5 g/L. For patients needing an invasive procedure soon after TPE, a full replacement with FFP is suggested [4]. Interestingly, therapeutic-dose anticoagulation was not considered a contraindication to lowering the fibrinogen threshold in the study by Wodayo et al. and appeared to be the principal predictor of bleeding complications in their cohort [53,54].

## 7. Fibrinogen Replacement Options and Target Levels

Fibrinogen kinetics are often altered in patients treated with TPE. Most patients have autoimmune disorders [4], many of which are associated with elevated baseline fibrinogen concentrations because fibrinogen acts as an acute-phase reactant [60,61]. At the same time, many of these conditions are treated with immunosuppressive therapy, which may substantially reduce hepatic fibrinogen synthesis [62,63,64]. Consequently, the combination of impaired fibrinogen production and abrupt fibrinogen removal during repeated TPE treatments may result in profound hypofibrinogenemia. There is a clinical impression that a proportion of patients have slower fibrinogen recovery. To our knowledge, no studies have specifically identified predictors of severe hypofibrinogenemia after initiation of TPE treatment or characteristics of patients with particularly slow fibrinogen recovery. Patients with pre-existing liver disease or with low baseline fibrinogen concentrations are likely at greatest risk.

The commonly used fibrinogen threshold of 1.0 g/L before TPE, as discussed above, is probably based on experience with congenital fibrinogen disorders, where prophylactic replacement therapy maintaining a trough fibrinogen concentration above 0.5 g/L is generally recommended to prevent spontaneous bleeding. Substantially higher concentrations are required in the presence of active bleeding or prior to surgery, usually between 1.5 and 2.0 g/L [65,66,67]. Similar thresholds are likely appropriate also in the apheresis setting for patients with increased bleeding risk in the peri-procedural setting, as discussed above.

Fibrinogen replacement may be achieved using FFP, cryoprecipitate, or fibrinogen concentrate [68]. FFP contains all coagulation factors and inhibitors, whereas cryoprecipitate is enriched predominantly with high-molecular-weight proteins, including fibrinogen and factor XIII. However, neither product has a standardized fibrinogen concentration; both require ABO compatibility, and both carry a residual risk of viral transmission [22,68]. Fibrinogen concentrates are generally considered a safer alternative, although they contain primarily fibrinogen with possible remnants of variable amounts of factor XIII, depending on the manufacturer [69,70,71]. Although some studies suggest potential advantages of fibrinogen concentrate over FFP or cryoprecipitate in trauma and other bleeding settings [72,73], these situations differ substantially from routine TPE treatment, where frozen plasma products can be thawed in advance and administered in isovolumic fashion. Nevertheless, compared with untreated plasma-derived products, fibrinogen concentrates are also associated with a lower risk of allergic reactions, alloimmunization, and citrate accumulation [5,74]. Therefore, fibrinogen concentrate might be the best option for fibrinogen replacement in apheresis in patients without very high bleeding risk. In our pilot study, fibrinogen supplementation was successfully achieved using both fibrinogen concentrate and FFP. No bleeding complications occurred in either group; however, ROTEM analyses suggested superior preservation of hemostatic potential in patients receiving FFP [20]. With active bleeding or very high bleeding risk, on the other hand, FFP should be used to ensure adequate levels of all clotting factors.

## 8. Possibility of Factor XIII Deficiency with Fibrinogen-Only Replacement

Fibrinogen has a relatively long half-life, and its plasma concentration is likely closely interconnected with factor XIII activity, which also declines substantially during repetitive TPE procedures. Indeed, Krämer et al. described a decrease in factor XIII activity comparable to the reduction in fibrinogen concentration following consecutive TPE treatments [41]. In their study, TPE procedures were performed at mean intervals of 2.7 days. Mean fibrinogen concentration before the sixth TPE decreased significantly to 1.07 g/L compared with 4 g/L before the first procedure. Similarly, factor XIII activity declined from 150% before the first TPE to 44% before the sixth procedure. Therefore, it is conceivable that with repeated fibrinogen-only replacement, fibrinogen levels might be maintained, but factor XIII could become severely reduced.

Minimal hemostatic levels of factor XIII are very low (2–5%), and factor XIII is also present within the thrombocytes [75], which might ameliorate its deficiency in the plasma. In our pilot study on fibrinogen-only replacement, we did not observe critical factor XIII levels after TPEs with fibrinogen concentrate replacement (post-TPE factor XIII levels were about 17%), and levels were comparable to post-TPE levels with albumin replacement, where patients had higher pre-TPE fibrinogen and factor XIII levels [20]. In a report from Chuliber et al., acquired factor XIII deficiency with TPE treatment was linked to bleeding episodes, although fibrinogen values were not reported during the bleeding episodes, and the majority of bleeding occurred after surgery [76]. Therefore, the clinical significance of factor XIII deficiency during TPE treatment remains to be established.

Furthermore, fibrinogen concentrates also contain variable amounts of factor XIII, depending on the manufacturer [69,70,71]. This variability may become clinically relevant during repetitive use of fibrinogen concentrate.

## 9. Fibrinogen-Sparing Apheresis Procedures

Another possibility for maintaining adequate fibrinogen levels is to use more selective apheresis techniques, which remove much less fibrinogen. One such technique is immunoadsorption, which selectively removes immunoglobulins. Nevertheless, fibrinogen is also partially removed and drops by about 30% during a single procedure, during which 2–3 plasma volumes are usually processed [49]. As the procedure is usually not performed very frequently, it is unlikely to induce profound hypofibrinogenemia or coagulopathy [77,78,79,80].

Double-filtration plasmapheresis (DFPF) is a procedure where plasma is filtered through a regular plasma filter, but then a secondary filter returns small plasma constituents to reduce the need for replacement solution. Removal of clotting factors depends on the characteristics of the secondary filter, with the majority of filters resulting in similar loss as TPE, while some have reduced removal down to 30%, similar to immunoadsorption [81].

Recently, a selective plasma exchange with the use of a selective plasma separator with smaller pores of 0.03 μm has been described. While its sieving coefficient for IgG has been shown to be above 0.5 (similarly to nonselective plasma exchange), the sieving coefficients for factor XIII (320 kDa) and fibrinogen (340 kDa) are only 0.17 and 0, with described reductions in factor XIII and fibrinogen after a single procedure of approximately 19% and 16%. Because it cannot remove larger molecules (for example, IgM), the procedure can be used when IgG removal is preferred [82,83].

## 10. Conclusions

Therapeutic apheresis procedures influence patients’ coagulation potential. Partial removal of coagulation factors without their replacement is a unique phenomenon in medicine that cannot be easily compared with other conditions. A combination of depletional and dilutional coagulopathy in an actively bleeding trauma patient or in a septic patient who is resuscitated with fluids and has a high and ongoing consumption of coagulation factors and thrombocytes, concomitant anemia and systemic inflammatory response syndrome probably cannot be completely compared with a reasonably stable patient on TPE treatment. Different recovery times of coagulation factors and inhibitors can cause an imbalance of their concentration ratios during treatment with TPE procedures, influencing both the risk for bleeding and thrombosis. While the required hemostatic levels of different factors for effective hemostasis in patients with inherited coagulation disorders can offer some guidance for bleeding prevention, patients with those disorders usually have low concentrations of only one coagulation factor, while TPE patients can have low levels of most factors. Patients with inherited coagulation disorders cannot replenish the deficient factor by themselves, but patients on TPE treatment eventually do recover factors if no further procedure is performed, so their risk for bleeding is temporary. It is hard to assess how dangerous this temporary bleeding risk is for every patient, as too many factors are involved. Additionally, patients’ susceptibility to thrombotic complications and recent or planned invasive procedures complicate this assessment even further. Management of concomitant anticoagulant therapy is also difficult.

Given fibrinogen’s long half-life, slow recovery time and central importance in hemostasis, it is probably the best routinely available marker to assess patients’ bleeding susceptibility as a consequence of repetitive TPE procedures. While some associations advise against the need for its monitoring during standard TPE treatment regimens, and there are reports describing no major bleeding episodes even with daily TPEs for up to five or even ten procedures, resulting in extremely low fibrinogen levels, it is the authors’ opinion that there is no need to walk right on the edge of the high bleeding risks for our patients, as it is safer to prevent the occurrence of bleeding than to treat it, especially as bleeding complications can have detrimental consequences. Furthermore, besides determining fibrinogen levels, the patients’ bleeding risk should also be assessed, focusing on concomitant diseases, medications and recent or planned invasive procedures. The proposed new “safe” preprocedural fibrinogen level of 0.8 g/L seems low and should not be used for patients on anticoagulant therapy or with bleeding risk. Fibrinogen concentrates probably represent a safe option for fibrinogen supplementation, but further studies are still needed.

## Figures and Tables

**Table 1:** Restitution of clotting factor levels reported as the percentage of occasions during a course of TPE on which the levels before TPE fell within the normal range, depending on the time interval between TPEs (as reported for 64 TPEs by Chirnside et al. exchanging plasma volume of 4000 mL [10]).

Coagulation Factor	After 1 Day	After 2 Days	After 3 or More Days
Fibrinogen	50%	52%	74%
Prothrombin	75%	87%	80%
V	90%	100%	100%
VII	95%	96%	95%
VIII	100%	100%	100%
IX	100%	100%	100%
X	30%	56% *	70% *
ATIII	85%	87%	100%

* for factor X almost complete restitution rates after 2–3 days are reported by Sultan et al. exchanging 160% of patients’ plasma volume [16].

**Table 2:** Hemostatic levels of different factors needed for effective hemostasis in patients with inherited coagulation disorders and their half-lives after intravenous replacement (as reported by Mannucci et al. [23]).

Deficient Factor	Hemostatic Levels	Plasma Half-Life After Replacement
Fibrinogen	0.5 g/L	2–4 days
Prothrombin	20–30%	3–4 days
V	15–20%	36 h
VII	15–20%	4–6 h
X	15–20%	40–60 h
XI	15–20%	40–70 h
XIII	2–5%	11–14 days

**Table 3:** Summary table of mentioned articles regarding bleeding episodes and thromboses.

Article	Number of Patients	Number of Procedures	Exchange Volume	Bleeding Episodes	Frequency of Bleeding Episodes	Reported Fibrinogen Values	Thrombotic Events
Sultan et al., 1979 [16]	7	18	160% plasma volume	0	0	>0.3 g/L after all TPEs	2 (1 associated with central venous catheter, 1 clotting in the centrifuge bowl)
Keller et al., 1979 [18]	18	179	4 or 5.2 L	3 (1 hemoptysis, 1 rectal bleeding, 1 epistaxis) + 3 episodes of prolonged oozing from cannulation sites	2.2% per procedure	Could be very low after daily TPEs for some patients. Was >1.3 g/L before and 0.33, 0.71 and 0.53 g/L after TPEs for bleeding patients, all of whom had concomitant thrombocytopenia.	NA
Ma et al., 2025 [47]	145	890	1 plasma volume	19 (1 fatal—after lung biopsy, 5 major)	8% of patients had clinically relevant bleeding	1.75 g/L before fatal bleeding event with concurrent thrombocytopenia. Fibrinogen and thrombocyte values for other cases are not reported; fibrinogen was not checked routinely.	8 (4%, 6 deep vein thromboses, 2 CVC-associated)
Soares Ferreira et al., 2023 [48]	779	NA	NA	135 patients	17.3% of patients	NA	NA
Zöllner et al., 2014 [49]	67	1032	1–1.5 plasma volume	18 events in 10 patients (3 major)	1.7% per procedure (TPE, TPE/IA or IA), 2.3% per TPE procedure	One of 62 post-treatment fibrinogen concentrations of <1 g/L resulted in a bleeding event (after IA); other bleeding events occurred with higher fibrinogen values.	NA
Shemin et al., 2007 [50]	174	1727	1 plasma volume	23 (21 of them only exit-site bleeding)	1.3% per procedure	NA	NA
Basic-Jukic et al., 2005 [51]	509	4857	1–2 plasma volumes	3 (2 prolonged oozing from exit sites, 1 epistaxis)	0.06% per procedure	NA	NA
Raval et al., 2026 [52]	228	1351	1 plasma volume	14 (13 of them CVC-associated)	1.4% per consecutive daily TPE procedure and 6.1% per patient	NA, but probably low (bleeding occurred after 3 or more consecutive TPEs)	NA
Wodajo et al., 2024 [53]	275	1406	1–1.2 plasma volume	4 (1 hematuria, 3 access-site-associated)	2% of patients	<1.5 g/L before TPE, on anticoagulant therapy	NA

Abbreviations: NA = not available; CVC = central venous catheter; TPE = therapeutic plasma exchange.

**Table 4:** Proposed stratification for procedural bleeding risk for patients needing anticoagulation treatment (taken from Spyropoulus et al. [57]).

High Bleeding Risk Procedures (≥2%)	Low Bleeding Risk Procedures (<2%)	Minimal Bleeding Risk Procedures
Major surgery with extensive tissue injury	Arthroscopy	Minor dermatologic procedures
Cancer surgery	Cutaneous/lymph node biopsies	Cataract procedures
Major orthopedic surgery	Shoulder/foot/hand surgery	Minor dental procedures
Reconstructive plastic surgery	Coronary angiography	Pacemaker/ICD implantation
Urologic or gastrointestinal surgery	Gastrointestinal endoscopy ± biopsy	
Transurethral prostate resection	Colonoscopy ± biopsy	
Nephrectomy, kidney biopsy	Abdominal hysterectomy	
Colonic polyp resection	Laparoscopic cholecystectomy	
Bowel resection	Abdominal hernia repair	
PEG, ERCP	Hemorrhoidal surgery	
Highly vascular organs surgery	Bronchoscopy ± biopsy	

ERCP—endoscopic retrograde cholangiopancreatography, ICD—implantable cardioverter-defibrillator, PEG—percutaneous endoscopic gastrostomy.

## Data Availability

No new data were created or analyzed in this study. Data sharing is not applicable to this article.
